# Clinicopathological impact of VEGFR2 and VEGF‐C in patients with *EGFR* ‐major mutant NSCLC receiving osimertinib

**DOI:** 10.1111/1759-7714.15082

**Published:** 2023-08-22

**Authors:** Kyoichi Kaira, Hisao Imai, Atsuto Mouri, Kosuke Hashimoto, Yu Miura, Ayako Shiono, Ou Yamaguchi, Kunihiko Kobayashi, Tomonori Kawasaki, Masanori Yasuda, Hiroshi Kagamu

**Affiliations:** ^1^ Department of Respiratory Medicine Saitama Medical University Hidaka city Japan; ^2^ Department of Pathology, International Medical Center Saitama Medical University Hidaka city Japan

## Abstract

**Background:**

Vascular endothelial growth factor (VEGF) has been identified as one of the resistant mechanisms to epidermal growth factor receptor‐tyrosine kinase inhibitors (EGFR‐TKIs). However, the relationship between the efficacy of osimertinib and protein expression of VEGF family members in patients with advanced non‐small cell lung cancer (NSCLC) harboring *EGFR* mutations remains unclear.

**Methods:**

A total of 76 patients with advanced NSCLC with *EGFR* major mutations (del19 or L858R) receiving first‐line osimertinib were eligible as the osimertinib (Osi) group, whereas 43 patients receiving first‐ or second‐generation EGFR‐TKIs were compared with the control group. The expression of vascular endothelial growth factor receptor 2 (VEGFR2) and vascular endothelial growth factor C (VEGF‐C) in the tumor specimens was analyzed using immunohistochemistry.

**Results:**

VEGFR2 and VEGF‐C were highly expressed in 65.8% and 51.3% of patients, respectively, in the Osi group, and 69.7% and 76.7%, respectively, in the control group. High VEGFR2 and VEGF‐C levels were significantly associated with poor performance status (PS) and female sex, respectively. In the Osi group, patients with co‐high expression of VEGFR2 and VEGF‐C showed significantly worse progression‐free survival (PFS) and overall survival (OS) than those without co‐high expression. In del19, VEGFR2 was a significant predictor of PFS and OS and independent predictor of OS in multivariate analysis. In L858R, co‐high expression of VEGFR2 and VEGF‐C was identified as a significant predictor of PFS and OS and independent predictor of PFS.

**Conclusion:**

VEGFR2 and VEGF‐C are highly expressed in *EGFR*‐mutant NSCLC cells. Increased expression of VEGFR2 was identified as a significant prognostic factor in patients with *EGFR* del19 mutation who received osimertinib, whereas co‐high expression of VEGFR2 and VEGF‐C was a significant predictor for those with *EGFR* L858R mutation.

## Graphical abstract

VEGF‐agonist and ligand are highly expressed in *EGFR*‐mutant NSCLC. Co‐high expression of VEGFR2 and VEGF‐C are related to worse prognosis for patients receiving osimertinib. High VEGFR2 expression was identified as a significant predictor in *EGFR* del19 mutation. Co‐high expression of VEGFR2 and VEGF‐C was a significant predictor for *EGFR* L858R mutation.

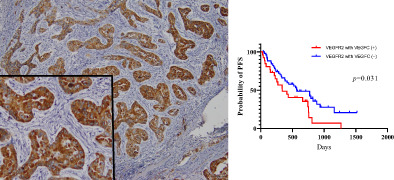

## INTRODUCTION

Epidermal growth factor receptor (*EGFR*) mutations are major oncogenic alterations in non‐small cell lung cancer (NSCLC), particularly adenocarcinoma, and have been identified as molecular targets.^1^ Currently, three types of *EGFR* tyrosine kinase inhibitors (TKIs) exist including first, second, and third generations available for *EGFR*‐mutant NSCLC.^2^, ^3^, ^4^ Although T790M is well known as an acquired resistance to first‐ or second‐generation EGFR‐TKIs, the mechanism of resistance to osimertinib as third‐generation EGFR‐TKI remains unclear. Many experimental and clinical studies have been conducted to overcome resistance to osimertinib treatment.

Recently, it has been reported that the expression of vascular endothelial growth factor receptor 2 (VEGFR2) is higher in EGFR‐TKI‐resistant cell lines than in TKI‐sensitive cell lines.^5^ In a previous study, *EGFR* activation via EGF or transforming growth factor‐alpha (TGF‐α) increased VEGF production.^6^ Watanabe et al. described that VEGF‐A was highly expressed in mutant *EGFR* cells and that the *EGFR* signaling pathway can induce VEGFR2 pathway activation, suggesting the enhancement of antitumor effects of EGFR‐TKI through VEGFR2 inhibition.^7^ A recent clinical trial demonstrated that ramucirumab (a VEGFR2 inhibitor) plus erlotinib achieved superior progression‐free survival (PFS) compared with erlotinib alone in untreated *EGFR*‐mutated NSCLC.^8^ Although the evidence of osimertinib plus VEGF inhibitors in *EGFR* mutated NSCLC remains unknown, a phase 1/2 trial revealed that the combination of osimertinib and bevacizumab met the primary endpoint of progression‐free survival at 12 months; thus, a randomized phase 3 trial comparing osimertinib plus bevacizumab and osimertinib is planned.^9^ In contrast, a phase 2 study failed to demonstrate the efficacy of osimertinib plus bevacizumab in improving PFS in patients with *EGFR*‐mutated NSCLC.^10^ Therefore, the therapeutic significance of VEGF inhibitors, in addition to EGFR‐TKIs, may differ between the first‐ or second‐ and third‐generation EGFR‐TKIs. Although the VEGF signaling pathway has been previously investigated as a potential EGFR‐TKI resistance pathway,^5^, ^6^, ^7^ it remains unclear whether the expression level of the VEGF‐ligand or VEGFR‐receptor could predict the outcome after EGFR‐TKI monotherapy in advanced *EGFR*‐mutated NSCLC. In particular, the efficacy of osimertinib and the expression of VEGF in tumor specimens have not been described. Our previous investigations confirmed that VEGF‐A, ‐B, and ‐D as VEGF‐ligand and VEGFR1 and 3 as VEGFR‐receptor were not suitable for immunohistochemistry in human tumor specimens because of nonspecific staining.^11^, ^12^ A recent study revealed that vascular endothelial growth factor C (VEGF‐C) is closely associated with EGFR‐TKI resistance in lung adenocarcinoma.^13^

Based on this background, we conducted a clinicopathological study to evaluate the prognostic impact of VEGF‐ligand and VEGFR‐receptor expression after osimertinib treatment in patients with advanced or metastatic *EGFR*‐mutated NSCLC, using Ki‐67 as a tumor cell proliferation marker and system alanine–serine–cysteine amino acid transporter‐2 (ASCT2) as amino acid transporter.

## METHODS

### Patients

Between August 2018 and July 2021, 105 patients with advanced or metastatic NSCLC harboring *EGFR* mutations were treated with osimertinib as a first‐line treatment in our institution. The inclusion criteria were a therapeutic history of first‐line osimertinib, major *EGFR* mutant tumors with deletions in exon 19 (del19) or exon 21 codon p.Leu858Arg (L858R), and sufficient tumor specimens for immunohistochemistry. Of these, six patients exhibited *EGFR* mutations (L861Q, G719X, T790M, exon 20 insertion, and S768I) without del19 or L858R, and 23 patients did not have sufficient tumor specimens for immunohistochemistry before osimertinib therapy. Therefore, 76 patients (n^male^ = 37, n^female^ = 39; median age = 70 years, range = 45–88 years) were eligible for this study (Osi group). Compared with the 76 patients receiving osimertinib, conversely, the patients with advanced NSCLC harboring major *EGFR* mutations (del19 or L858R) who received first‐ or second‐generation EGFR‐TKI were also analyzed as a control group. Forty‐three patients were eligible for further analysis between September 2011 and November 2018. 2. Most patients in the control group were definitively diagnosed by cytological examination; thus, recruiting more patients was difficult owing to the lack of tumor specimens. *EGFR* mutation testing using Cobas was performed according to the manufacturer's protocol.

Clinical data, such as age, sex, performance status (PS), smoking history, radiological examination, and survival information, were extracted from the medical records. This study was approved by the Institutional Ethics Committee of the International Medical Center of Saitama Medical University (approval no. 19‐075), Hidaka City, Japan. The Ethical Committee waived the need to obtain written informed consent for the use of human tissues to participate from the patients owing to the retrospective nature of the study.

### Therapeutic evaluation

Osimertinib (80 mg/day) was orally administered as first‐line treatment in 76 patients. In the control group, gefitinib 250 mg/day, erlotinib 150 mg/day, or afatinib 30 mg/day as first‐ or second‐generation EGFR‐TKI was orally administered. Physical examinations were performed and complete blood counts, biochemical tests for liver and renal dysfunction, electrolytes, and adverse events were examined by the chief physician. Toxicity was graded based on the Common Terminology Criteria for Adverse Events, version 4.0.^14^ Tumor response was examined based on the Response Evaluation Criteria in Solid Tumors version 1.1.^15^ Objective response rate (ORR) and disease control rate (DCR) were assessed. DCR was defined as the percentages of complete response (CR), partial response (PR), or stable disease (SD).

### Immunohistochemical staining

Immunohistochemical staining was performed as previously described.^12^, ^13^ VEGFR2 rabbit polyclonal antibody (1:100; Cell Signaling Technology, Inc.; cat #2472) and VEGF‐C rabbit polyclonal antibody (1:20; Immuno‐Biological Laboratories Company Ltd) were scored based on the stained tumor area (biopsy and surgical samples) as follows: 1, ≤10; 2, 11–24; 3, 25–49; and 4, ≥50% staining. Low and high expression levels were defined as scores of 1–3 and 4 for VEGFR2 and VEGF‐C, respectively, as previously described.^12^, ^13^ If no expression was detected, a score of 0 was defined. Ki‐67 mouse monoclonal antibody (MIB‐1, 1:100 Dako; M7240) and ASCT2 rabbit monoclonal antibody (D7C12, 1:100 Cell Signaling Technology, Inc #8057) were quantified based on previous procedures.^12^ High and low expression levels were defined as the median values for each marker. The median values of VEGFR2, VEGF‐C, ASCT2 and Ki‐67 were 4 (score 1 to 4), 4 (score 1 to 4), 430 (0 to 972), and 7.5 (0 to 399), respectively.

Sections were evaluated by at least two researchers (KK and HI) using a light microscope (×200 and ×400 magnification) in a blinded fashion. In the case of discrepancies, both investigators evaluated the slides simultaneously until a final consensus was reached. The investigators were blinded to the patient outcomes.

### Statistical analysis

Student's *t* (unpaired *t*) and *χ* ^2^ tests were used for continuous and categorical variables, respectively. Statistical significance was set at *p* < 0.05. PFS was defined as the time from initial EGFR‐TKI treatment to disease progression or death. Overall survival (OS) was defined as the time from the initial EGFR‐TKI treatment to death from any cause. The Kaplan–Meier method was used to estimate survival as a function of time, and survival differences were analyzed using the log‐rank test. Univariate and multivariate analyses were performed using logistic regression. All statistical analyses were performed using GraphPad Prism (version 8.0; GraphPad Software, Inc.) and JMP 14.0 (SAS Institute, Inc.).

## RESULTS

### Patient demographics

Patient characteristics based on VEGFR2 and VEGF‐C expression are listed in Table 1. In the 76 patients (Osi group), the PS was 0, 1, 2, 3, and 4 in 32 (42.1%), 32 (42.1%), 7 (9.2%), 3 (4.0%), and 2 (2.6%) patients, respectively. A smoking history was observed in 30 (39.5%) patients. *EGFR* mutation status, del19 and L858R were detected in 39 (51.3%) and 37 (48.7%) patients, respectively.

**TABLE 1:** Patient demographics based on VEGFR2 and VEGF‐C expression.

Different variables		Total number	VEGFR2			VEGF‐C		
		*N* = 76	High (*n* = 50)	Low (*n* = 26)	*p*‐value	High (*n* = 39)	Low (*n* = 37)	*p*‐value
Age	<75/≥75 yrs	48/28	34/16	14/12	0.316	19/20	29/8	**0.009**
Gender	Male/female	37/39	22/28	15/11	0.334	16/23	21/16	0.294
ECOG PS	0–1/2–4	64/12	39/11	25/1	**0.049**	31/8	32/5	0.525
Smoking	Yes/No	30/46	21/29	9/17	0.624	15/24	15/22	>0.999
Disease stage	IV/Ope rec.	59/17	42/8	17/9	0.084	27/12	32/5	0.099
Mutation	Del 19/L858R	39/37	27/23	12/14	0.629	17/22	22/15	0.178
CNS meta.	Yes/No	32/44	24/26	8/18	0.221	18/21	14/23	0.494
PM	Yes/No	25/51	18/32	7/19	0.454	12/27	13/24	0.807
Pleural ca.	Yes/No	31/45	18/32	13/13	0.325	14/25	17/20	0.484
Liver meta.	Yes/No	7/69	5/45	2/24	>0.999	4/35	3/34	>0.999
Bone meta.	Yes/No	33/43	25/25	8/18	0.145	17/22	16/21	>0.999
Ki‐67 LI	High/Low	38/38	25/25	13/13	0.085	19/20	19/18	>0.9990
ASCT2	High/Low	30/46	16/34	14/12	0.085	13/26	17/20	0.355

Abbreviations: CNS, central nervous system; ECOG PS, Eastern Cooperative Oncology group; LI, labeling index; meta, metastasis; Ope rec, recurrence after operation; PM, pulmonary metastases; VEGF‐C, vascular endothelial growth factor C; VEGFR2, vascular endothelial growth factor receptor 2.

The demographics of the 43 patients (n^male^ = 18, n^female^ = 25; age, median = 70 years, range = 38–86 years) in the control group are shown in Table A1 (online only). PS was 0, 1, 2, and 3 in 24 (55.8%), 11 (25.6%), 3 (7.0%), and five (11.6%) patients, respectively. Del19 and L858R mutations were observed in 32 (74.4%) and 11 (25.6%) patients, respectively. As therapeutic regimens for first‐ or second‐generation EGFR‐TKI, gefitinib, erlotinib, and afatinib were orally administered to 11 (25.6%), three (6.9%), and 29 (67.5%) patients, respectively.

Next, the patient demographics were compared between the Osi and control groups (Table A2, online only). Although there was a good balance in patient characteristics between both groups, the frequency of *EGFR* del19 mutations was significantly higher in the control than in the Osi group.

### Immunohistochemistry for angiogenetic marker

**FIGURE 1:**
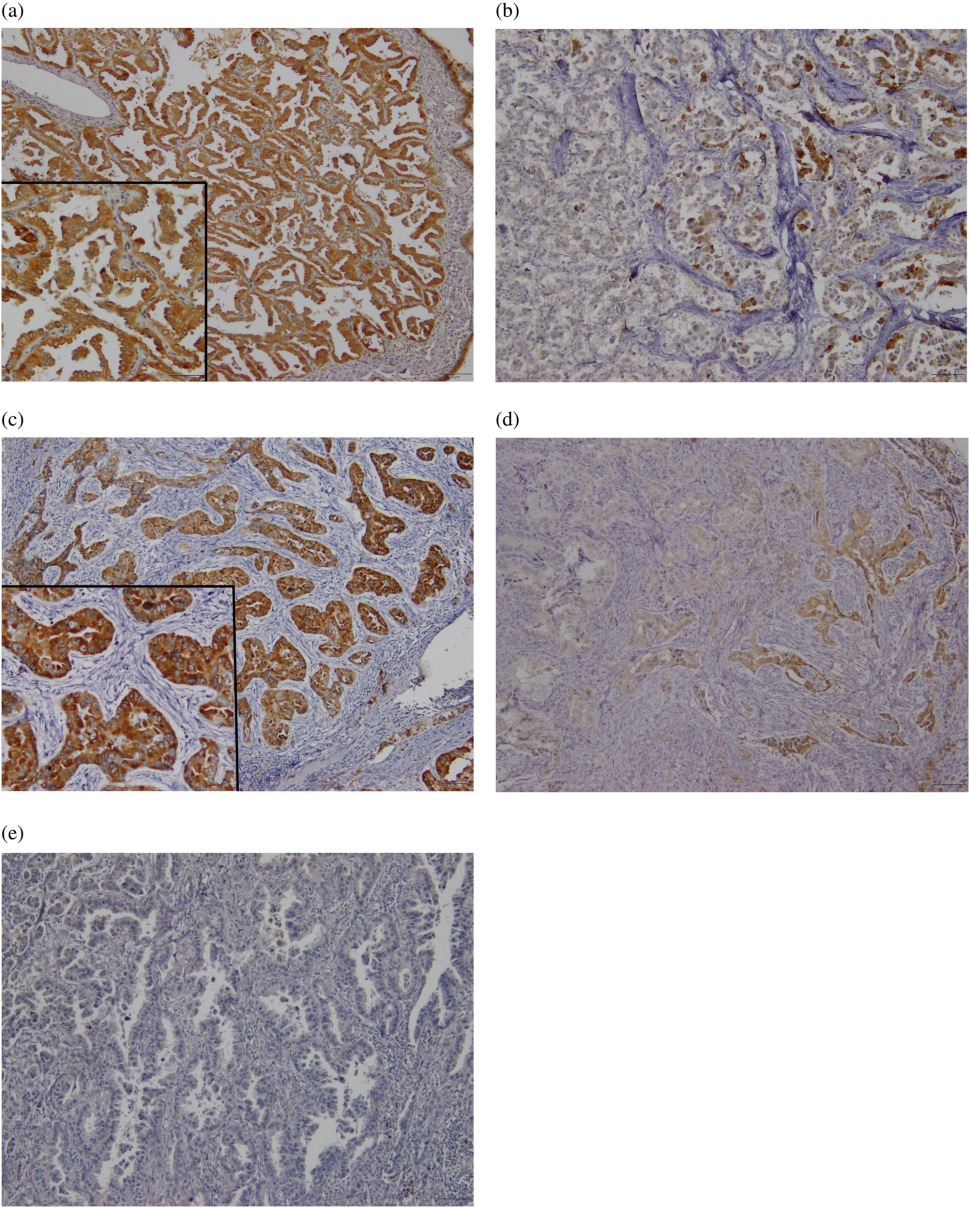
Immunohistochemistry staining for vascular endothelial growth factor receptor 2 (VEGFR2) and VEGF‐C expression in non‐small cell lung cancer. VEGFR2 and VEGF‐C were strongly stained on cell membranes and cytoplasm of tumor specimens. Representative images of 4 (a) and 2 (b) for VEGF‐C expression scores, and 4 (c), 3 (d) and 1 (e) for VEGFR2 expression scores. Low magnification, x200; high magnification, x400. VEGFR, vascular endothelial growth factor receptor; VEGF‐C, vascular endothelial growth factor C.

Immunohistochemical staining for VEGFR2 and VEGF‐C was performed on all tumor tissues. Biopsy and surgical specimens for immunohistochemistry were obtained from 59 (77.6%) and 17 (22.4%) patients, respectively, for the Osi group and 26 (60.5%) and 17 (39.5%) patients, respectively, for the control group. Representative images of VEGFR2 and VEGF‐C expression are shown in Figure 1. Immunostaining for VEGFR2 and VEGF‐C was performed on the cell membranes and cytoplasm of tumor specimens. The frequencies of high VEGFR2 expression in the Osi‐ and control groups were 65.8% (50/76) and 69.7% (30/43), respectively (*p* = 0.689), and those of high VEGF‐C expression in the Osi and control groups were 51.3% (39/76) and 76.7% (33/43), respectively (*p* = 0.006). In the Osi group, the incidences of scores 1, 2, 3, and 4 were 0 (0.0%), 5 (6.6%), 21 (27.6%), and 50 (65.8%), respectively, for VEGFR2, and 4 (5.3%), 10 (13.1%), 23 (30.3%), and 39 (51.3%), respectively, for VEGF‐C. The patients with no expression for VEGFR2 and VEGF‐C were not observed. High VEGFR2 expression was significantly related to poor PS, and high VEGF‐C expression was closely associated with elderly age (Table 1).

### Efficacy based on angiogenetic markers

Table 2 and Table A3 (online only) show the therapeutic efficacy based on VEGFR2 and VEGFC expression. The ORR and DCR were 69.1% and 92.6%, respectively, for the Osi group, and 60.5% and 76.3%, respectively, for the control group. No statistically significant differences in the ORR and DCR were observed between patients with high and low VEGFR2 and VEGF‐C levels in the Osi and control groups.

**TABLE 2:** Efficacy according to VEGFR2 and VEGF‐C expression.

Response	All patients	Del 19	L858R	VEGFR2		VEFF‐C	
	(*n* = 68)	(*n* = 33)	(*n* = 35)	High (*n* = 46)	Low (*n* = 22)	High (*n* = 34)	Low (*n* = 34)
CR	2	1	1	1	1	1	1
PR	45	23	22	30	15	22	23
SD	16	7	9	12	4	7	9
PD	5	2	3	3	2	4	1
ORR (95% CI)	69.1% 57.3%–78.8%	72.7% 55.6%–85.1%	65.7% 49.1%–79.2%	67.3%	72.7%	67.6%	70.65%
				*p* = 0.782		*p* > 0.999	
DCR (95% CI)	92.6% 83.5%–97.2%	93.9% 79.4%–99.3%	91.4% 76.8%–97.8%	93.4%	90.9%	88.2%	97.1%
				*p* = 0.655		*p* = 0.355	

Abbreviations: 95% CI, 95% confidence interval; CR, complete response; DCR, disease control rate; ORR, objective response rate; PR, partial response; PR, progressive disease; SD, stable disease; VEGF‐C, vascular endothelial growth factor C; VEGFR2, vascular endothelial growth factor receptor 2.

Moreover, the ORR and DCR between del19 and L858R were not significantly different based on VEGFR2 and VEGF‐C expression.

### Survival analysis

**FIGURE 2:**
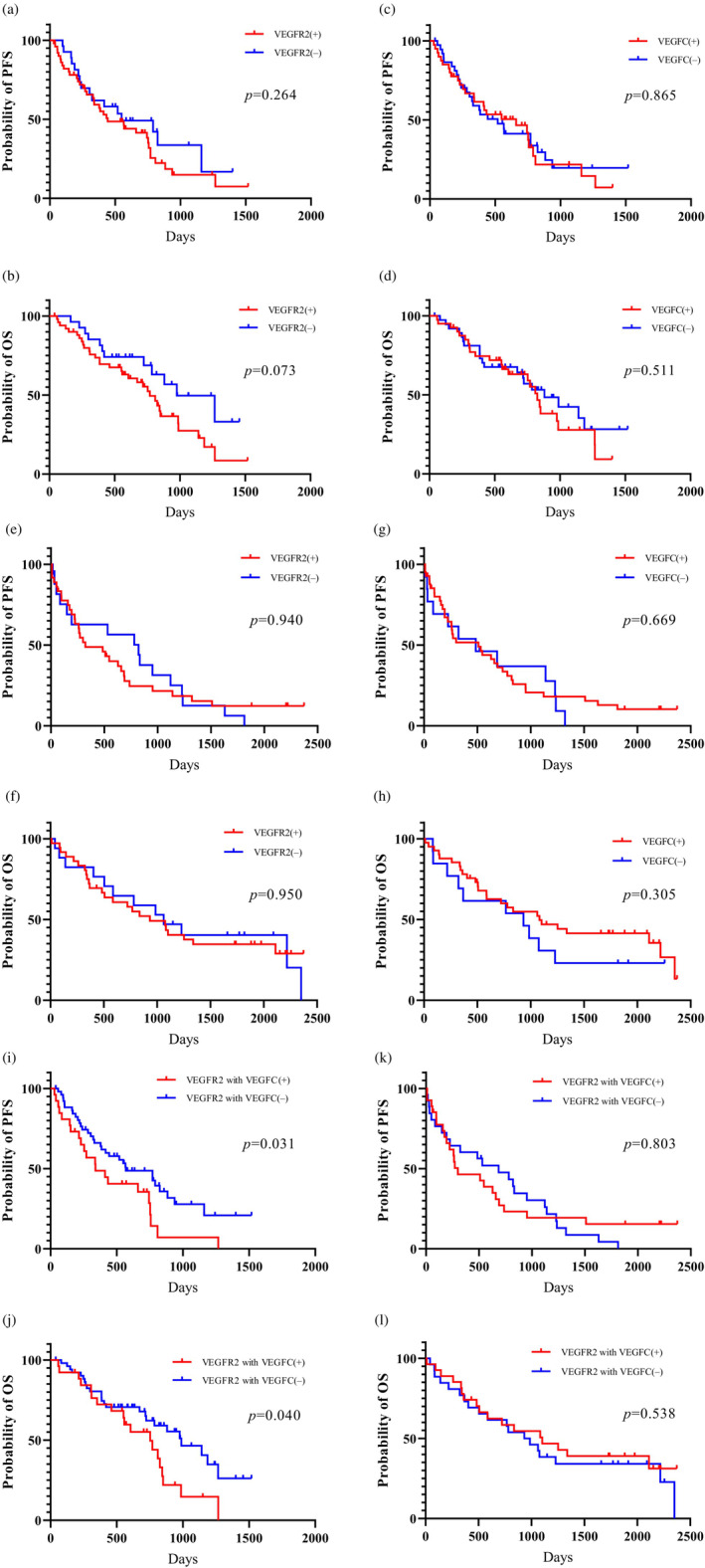
Kaplan–Meier survival curves of progression‐free survival (PFS) and overall survival (OS) based on vascular endothelial growth factor C (VEGF‐C) and vascular endothelial growth factor receptor 2 (VEGFR2) expression. In 76 patients receiving first‐line osimertinib, there was no statistically significant difference in PFS (a, c) and OS (b, d) according to VEGFR2 and VEGF‐C expression. In 43 patients treated with first‐ or second generation EGFR‐TKIs (control group), no statistically significance in PFS (e, g) and OS (f, h) was observed between the patients with high and low VEGFR2 and between those with high and low VEGF‐C expression. Coexpression of high VEGFR2 and VEGF‐C was identified as significantly worse PFS (I) and OS (J) in patients with osimertinib, but not in the control group (K, L). VEGFR2 with VEGF‐C (+) means coexpression of high VEGFR2 and VEGF‐C. VEGFR2 with VEGF‐C (−) means coexpression with low VEGFR2 and VEGF‐C or lack of coexpression.

**FIGURE 3:**
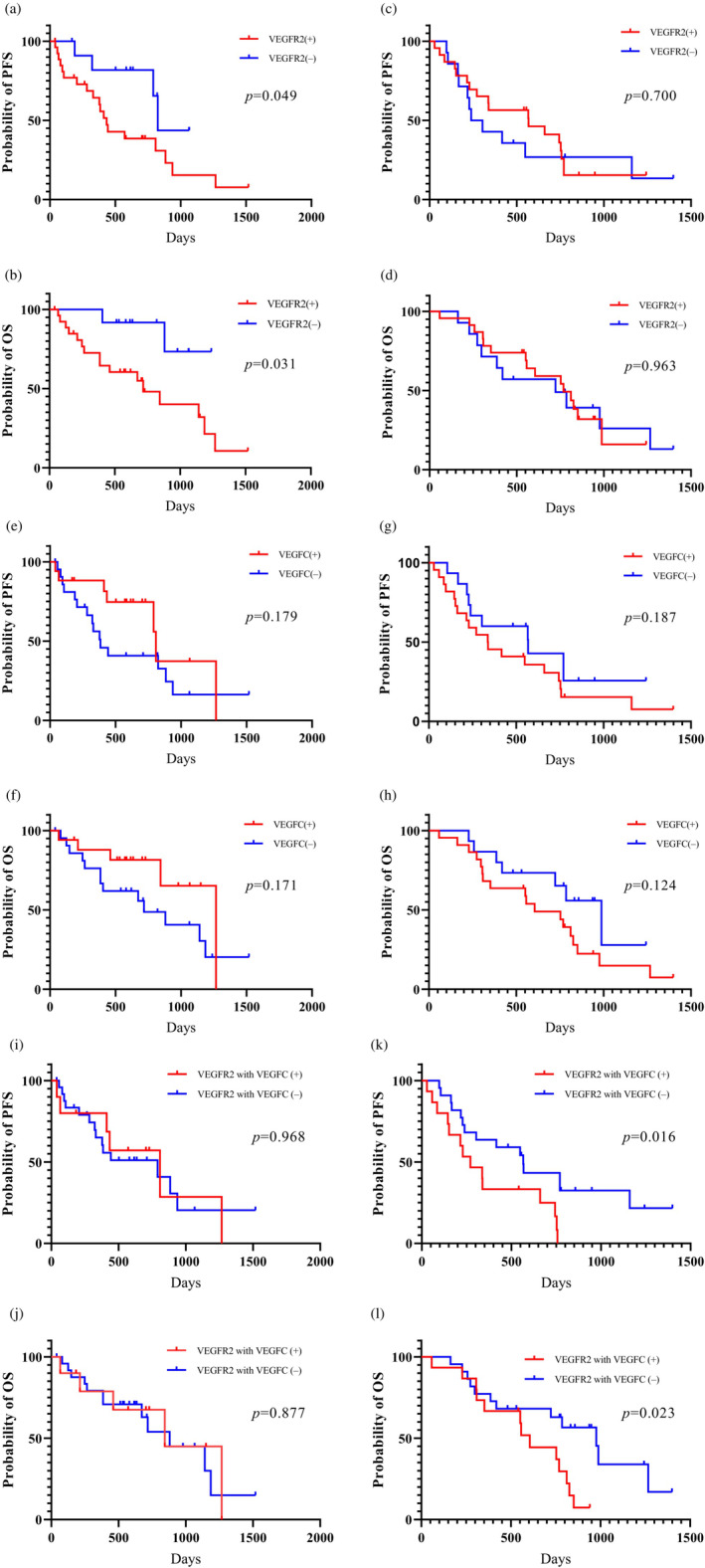
Kaplan–Meier survival curves of progression‐free survival (PFS) and overall survival (OS) according to del19 and L858R *EGFR* mutations status in 76 patients receiving osimertinib. All patients harboring del19 *EGFR* mutation with high vascular endothelial growth factor receptor 2 (VEGFR2) expression displayed a significant worse PFS (a) and OS (b), but not L858R *EGFR* mutation (c, d). No statistically significant difference in PFS (e, g) and OS (f, h) was observed all patients between high and low vascular endothelial growth factor C (VEGF‐C) expression. Coexpression of high VEGFR2 and VEGF‐C was identified as significantly worse PFS (i) and OS (j) in patients with L858R *EGFR* mutation, but not in those with del19 *EGFR* mutation (k, l). VEGFR2 with VEGF‐C (+) means coexpression of high VEGFR2 and VEGF‐C. VEGFR2 with VEGF‐C (−) means coexpression with low VEGFR2 and VEGF‐C or lack of coexpression.

In the Osi group, the median PFS and OS were 566 and 826 days, respectively. Fifty‐one patients experienced tumor recurrence, and 43 died due to progressive disease. In the control group, the median PFS and OS were 531 and 1082 days, respectively. A total of 37 patients experienced tumor recurrence, and 28 died because of progressive disease. Kaplan–Meier survival curves based on VEGFR2 and VEGF‐C expression were constructed in the Osi and control groups (Figure 2 and Figure 3). No statistically significant differences in PFS and OS were observed between patients with high and low VEGFR2 levels (Figure 2a,b) and between those with high and low VEGF‐C levels (Figure 2c,d) in the Osi group. Moreover, there was no significant difference in PFS and OS based on VEGFR2 (Figure 2e,f) and VEGF‐C (Figure 2g,h) in the control group. However, patients with coexpression of high VEGFR2 and VEGF‐C showed significantly worse PFS and OS than those with coexpression of low VEGFR2 and VEGF‐C or lack of coexpression (Figure 2i,j) in the Osi group, but not the control group (Figure 2k,l).

Next, survival analysis based on *EGFR* mutation status (del19 and L858R) was performed in both groups. In the Osi group, the PFS and OS based on del19 *EGFR* mutation were significantly worse in the patients with high VEGFR2 expression than in those with low VEGFR2 expression (Figure 3a,b), but not based on L858R (Figure 3c,d). No statistically significant differences in PFS and OS based on del19 (Figure 3e,f) and L858R (Figure 3g,h) *EGFR* mutations were observed between patients with high and low VEGF‐C expression. Patients harboring L858R with coexpression of high VEGFR2 and VEGF‐C displayed significantly worse PFS and OS than those with coexpression of low VEGFR2 and VEGF‐C or lack of coexpression (Figure 3k,l), but not with del19 (Figure 3i,j).

Univariate and multivariate survival analyses were performed based on the VEGFR2 and VEGF‐C expression levels in the Osi group (Table 3). Univariate analysis demonstrated worse PS and co‐high expression of VEGFR2 and VEGF‐C for PFS and PS as significant predictors for OS (Table 3). PS and the coexpression of VEGFR2 and VEGF‐C were selected for further multivariate analyses. Multivariate analysis confirmed that PS and coexpression of VEGFR2 and VEGF‐C were independent prognostic factors for PFS, and PS was a significant predictor of OS (Table 3). Next, a subanalysis based on different *EGFR* mutations with del19 (Table A4, online only) and L858R (Table A5, online only) was performed. In 39 patients with del19, univariate analysis revealed that PS and VEGFR2 were significant predictors for PFS and OS. Multivariate analysis confirmed that PS was an independent prognostic factor for PFS, and VEGFR2 was a significant predictor of OS (Table A4, online only). In 27 patients with L858R, the coexpression of VEGFR2 and VEGF‐C was identified as a significant predictor for PFS and OS, but not PS for OS. Multivariate analysis confirmed that the coexpression of VEGFR2 and VEGF‐C was an independent predictor for PFS, but not for OS (table 5, online only).

**TABLE 3:** Univariate and multivariate analysis in all patients (*n* = 76).

Different variables		Progression‐free survival					Overall survival				
		Univariate analysis		Multivariate analysis			Univariate analysis		Multivariate analysis		
		MST (days)	*p*‐value	HR	95% CI	*p*‐value	MST (days)	*p*‐value	HR	95% CI	*p*‐value
Age	<75/≥75 yrs	549/744	0.793	1.023	0.563–1.816	0.936	880/753	0.125	1.599	0.851–2.974	0.142
Gender	Male/Female	757/416	0.373	1.003	0.542–1.847	0.990	753/1142	0.142	1.061	0.524–2.160	0.867
ECOG PS	0–1/2–4	744/210	**0.001**	0.383	0.188–0.831	**0.016**	880/253	**<0.001**	0.333	0.149–0.783	**0.013**
Smoking	Yes/No	757/434	0.784				826/811	0.969			
Mutation	Del19/L858R	790/416	0.145				1142/769	0.175			
CNS meta.	Yes/No	549/744	0.227				753/850	0.130			
PM	Yes/No	397/660	0.221				785/844	0.218			
Pleural ca.	Yes/No	416/660	0.411				785/84	0.205			
Lever meta.	Yes/No	271/568	0.192				557/844	0.190			
Bone meta.	Yes/No	443/744	0.325				785/880	0.373			
Ki‐67 LI	High/Low	443/660	0.959				785/850	0.397			
ASCT2	High/Low	753/549	0.286				850/769	0.371			
VEGFR2	High/Low	443/790	0.251				769/976	0.108			
VEGF‐C	High/Low	660/443	0.889				811/880	0.614			
VEGFR2/VEGF‐C	Positive/Negative	412/568	**0.037**	1.563	0.821–2.900	0.170	753/976	**0.032**	1.428	0.689–2.867	0.329

Abbreviations: ASCT2, alanine–serine–cysteine amino acid transporter‐2; 95% CI, 95% confidence interval; CNS, central nervous system; ECOG PS, Eastern Cooperative Oncology Group; HR, hazard ratio; LI, labeling index; meta, metastasis; MST, median survival time; Ope rec, recurrence after operation; PM, pulmonary metastases; VEGF‐C, vascular endothelial growth factor C; VEGFR2, vascular endothelial growth factor receptor 2.

### Subsequent treatment

In the Osi group (*n* = 76), 26 (34.2%) patients were treated with sequential therapy after failure of osimertinib. Of these 26 patients, 15 patients received chemoimmunotherapy such as carboplatin, paclitaxel, bevacizumab and atezolizumab, six patients were treated with platinum‐based chemotherapy such as carboplatin plus pemetrexed or carboplatin plus nab‐paclitaxel, and five patients underwent first‐ or second‐generation EGFR‐TKIs such as gefitinib or afatinib. In the control group (*n* = 43), on the other hand, 22 (51.2%) patients were treated with sequential therapy. Of these 22 patients, seven received carboplatin plus pemetrexed, 12 patients were treated with osimertinib for the detection of T790M, and three patients underwent pemetrexed alone.

## DISCUSSION

This is the first study to evaluate the prognostic significance of VEGF‐C/VEGFR2 expression after osimertinib in patients with major *EGFR*‐mutated NSCLC. Interestingly, we found that the increased expression of VEGFR2 was a significant predictor of outcome in *EGFR* del19 mutation, whereas the co‐high expression of VEGFR2 and VEGF‐C was closely associated with a worse outcome in *EGFR* L858R mutation. Conversely, resistance to osimertinib is not closely associated with tumor cell proliferation and amino acid metabolism. Although it remains unknown why the therapeutic role of VEGF‐ligand and ‐receptor differed based on the major *EGFR* mutation status, further investigation is warranted to elucidate the different mechanisms between *EGFR* del19 and L858R mutations.

Two studies reported that osimertinib could significantly inhibit the expression of serum VEGF‐A, VEGF‐B, and VEGF‐C after treatment.^16^, ^17^ These studies suggest that the expression levels of serum VEGF‐ligand is associated with the therapeutic efficacy of osimertinib.^16^ Yuan et al. described that the expression of VEGF‐A, VEGFR1, and VEGFR2 was analyzed using immunohistochemistry in 104 patients with *EGFR*‐mutated lung adenocarcinoma who did not receive EGFR‐TKIs.^18^ In their study, low levels of VEGFR1 expression were more likely to be related to *EGFR* 19 exon mutations, and high‐level co‐expression of VEGF‐A and VEGFR1 was associated with *EGFR* 21 exon mutations, but not with VEGFR2.^18^ Although the relationship between the expression of angiogenic markers and outcome after EGFR‐TKIs remains unknown, high levels of VEGF‐ligand and VEGF‐receptor may play a crucial role in the pathogenesis of *EGFR* L858R mutations. Our study also identified co‐high expression of VEGFR2 and VEGF‐C as a significant predictor of outcome after osimertinib treatment without an explanation of the detailed mechanism. In our study, VEGFR2 was a significant predictor of osimertinib in patients with *EGFR* del19 mutations, unlike in a previous study.^18^ Several studies have demonstrated that increased VEGFR2 expression is a significant predictor of poor outcome in NSCLC.^5^, ^11^ Therefore, VEGFR2 expression may be associated with poor outcomes regardless of the induction of EGFR‐TKIs. Therefore, a large‐scale study is warranted to confirm our results. In addition, the therapeutic response in our study was not different between the Osi and control groups, and between *EGFR* del19 and L858R mutations. Scientifically, the outcome and response of third‐generation EGFR‐TKI such as osimertinib are known to be almost similar to those of first‐ or second‐generation EGFR‐TKIs. The therapeutic evaluation of our study correspond to previous approaches.^3^, ^4^, ^8^

VEGF family members, including VEGF‐A, ‐B, ‐C, VEGF‐D, and VEGF‐A, are key regulators of blood vessel development; VEGF‐B is related to embryonic angiogenesis, whereas VEGF‐C and VEGF‐D regulate lymphatic angiogenesis.^19^ As a possible resistance mechanism to *EGFR* mutant NSCLC, the upregulation of the *EGFR* signal pathway increased VEGF through hypoxia‐independent mechanisms; then the overexpression of VEGF family members plays a resistant role to EGFR‐TKIs.^19^ Therefore, the inhibition of the dual *EGFR* and VEGF pathways has been expected to lead to the activation of acquired EGFR‐TKI resistance. In our study, VEGF‐A and –B were not analyzed using immunohistochemistry because of the highly nonspecific staining, unlike the detection of serum levels. Although immunohistochemical analysis was attempted on VEGF‐A and ‐B, the results of these examinations were uncertain for further analysis. Therefore, VEGF‐A and ‐B were excluded from the analysis. Based on the results of our study, we hypothesized that VEGFR2 is associated with resistance to *EGFR* del19 mutations, whereas a combined increase in VEGFR2 and VEGF‐C contributes to the resistance of *EGFR* L858R. The signaling pathways of VEGF family members may differ based on the *EGFR* mutation status.

Owing to the therapeutic limitations of VEGF inhibitors with EGFR‐TKIs in previous studies, the combined regimen significantly improved PFS compared with EGFR‐TKIs alone; however, no available data show significantly prolonged OS.^8^, ^19^, ^20^ In our control group, VEGFR2 and VEGF‐C were highly expressed. However, utilizing the protein expression of VEGF within tumor tissues as a significant biomarker for outcome after first‐ or second‐generation EGFR‐TKI may be difficult. Conversely, in the Osi group, the prognostic role of VEGFR2 and VEGF‐C expression was slightly different based on *EGFR* mutation status; therefore, information regarding the protein expression of VEGF within tumor cells may be useful to suppress resistance to osimertinib for the choice of add‐on VEGF inhibitors. Overall, VEGF was highly expressed in the tumor specimens with *EGFR* mutations. However, we found that the VEGF family may play individual roles in resistance to EGFR‐TKIs based on different *EGFR* mutation statuses. In this study, we focused on the major *EGFR* mutations with del19 and L858R. When investigating uncommon mutations, such as G719X, or compound mutations, the protein expression of the VEGF family may exhibit different resistance to EGFR‐TKIs. Further clinical trials are warranted to evaluate the efficacy of osimertinib plus VEGF inhibitors based on the expression levels of the VEGF family.

Our study had several limitations. First, our sample size was small and limited, which may have biased the results. A larger sample size is necessary to confirm our conclusions. Second, the sample size of the control group was too small compared with that of the Osi group. Most patients who received first‐ or second‐generation EGFR‐TKI did not have sufficient tumor specimens for further immunohistochemistry. Thus, the prognostic significance of VEGFR2 and VEGF‐C expression in the control group remains unclear. Our control group was a relatively small sample size compared to the Osi group, thus, the difference of sample size may affect the lack of significant prognostic implication of VEGFR2 and VEGF‐C expression in control group. Finally, the crosstalk between the *EGFR* and VEGF signaling pathways should be discussed. It would be interesting to determine whether *EGFR* and VEGFR2 signaling can trigger the PI3K/AKT and RAS/RAF/ERK pathways, and whether the activation of *EGFR* can upregulate HIF‐1α, leading to VEGF gene expression. Our study lacked information on these signaling pathways and HIF‐1α expression because of the insufficient number of tumor specimens available for immunohistochemistry. Further investigations should focus on signaling pathways related to *EGFR* and VEGF.

In conclusion, VEGFR2 and VEGF‐C are highly expressed in patients with advanced NSCLC harboring *EGFR* mutations. Increased expression of VEGFR2 was identified as a significant prognostic factor in patients with *EGFR* del19 mutation who received osimertinib, whereas co‐high expression of VEGFR2 and VEGF‐C was a significant predictor for those with *EGFR* L858R mutation. Further combination therapy of osimertinib with a VEGF inhibitor may be considered an add‐on for VEGF inhibitors based on the protein expression levels of VEGFR2 and VEGF‐C within tumor specimens.

## AUTHOR CONTRIBUTIONS

Kyoichi Kaira, Ou Yamaguchi and Tomonori Kawasaki conceived the study and wrote the manuscript. Atsuto Mouri, Hiroshi Kagamu, Yu Miura, Ayako Shiono, Hisao Imai, Kunihiko Kobayashi and Masanori Yasuda collected and analyzed data. Kyoichi Kaira, Hisao Imai and Atsuto Mouri interpreted data. Kyoichi Kaira, Atsuto Mouri, Tomonori Kawasaki and Hiroshi Kagamu revised the manuscript. All authors read and approved the final manuscript. Kyoichi Kaira and Hisao Imai confirm the authenticity of all the raw data.

## FUNDING INFORMATION

The present study was supported by the Japan Society for the Promotion of Science (grant nos. 20K08118 and 21K07627).

## CONFLICT OF INTEREST STATEMENT

Kyoichi Kaira has received research grants and a speaker honorarium from Ono Pharmaceutical Company, Boehringer Ingelheim, Chugai Pharmaceutical, Taiho Pharmaceutical, Eli Lilly Japan and AstraZeneca. Atsuto Mouri and Ou Yamaguchi received a speaker honorarium from Eli Lilly, Taiho Pharmaceutical, Pfizer, Chugai Pharmaceutical and AstraZeneca. Hiroshi Kagamu has received research grants and a speaker honorarium from Ono Pharmaceutical Company, Bristol‐Myers Company, Boehringer Ingelheim, MSD, Daiichi Sankyo Company, Chugai Pharmaceutical, Taiho Pharmaceutical, Merck Biopharma Company, Eli Lilly Japan and AstraZeneca. Hisao Imai has received research grants and a speaker honorarium from Ono Pharmaceutical Company, AstraZeneca and Bristol‐Myers Company.

## ETHICS STATEMENT

The present study was approved by the Institutional Ethics Committee of the International Medical Center of Saitama Medical University, Hidaka city, Japan. The Ethical Committee waived the need to obtain written informed consent for the use of human tissues to participate from the patients owing to the retrospective nature of the study.

## Supporting information


**Table A1.** Patient demographics based on VEGFR2 and VEGF‐C expression (Control group).

**Table A2.** Comparison of patient demographics between Osi‐group and Control group.

**Table A3.** Efficacy according to VEGFR2 and VEGF‐C expression.

**Table A4.** Univariate and multivariate analysis in patients with del19 (*n* = 39).

**Table A5.** Univariate and multivariate analysis in patients with L858R (*n* = 27).

## Data Availability

The datasets generated and/or analyzed during the present study are available from the corresponding author on reasonable request.
