# Supplementary material for: Clinicopathological impact of VEGFR2 and VEGF‐C in patients with EGFR ‐major mutant NSCLC receiving osimertinib
Source: Thorac Cancer. 2023 Aug 22;14(29):2950–61. doi: 10.1111/1759-7714.15082 (PMC10569903; doi:10.1111/1759-7714.15082)
Supplement: Supplementary file 1 — Table A1. Patient demographics based on VEGFR2 and VEGF‐C expression (Control group). [file TCA-14-2950-s004.docx]

**Table A1. Patient’s demographics based on VEGFR2 and VEGF-C expression (Control group)**

| Different variables | | Total number | VEGFR2 | | | VEGF-C | | |
| --- | --- | --- | --- | --- | --- | --- | --- | --- |
|  |  | N=43 | High (n=30) | Low (n=13) | *p*-value | High (n=33) | Low (n=10) | *p*-value |
| Age | <75 / ≥75yrs | 30 / 13 | 21 / 9 | 9 / 4 | >0.999 | 23 / 10 | 7 / 3 | >0.999 |
| Gender | Male / Female | 18 / 25 | 12 / 18 | 6 / 7 | 0.746 | 15 / 18 | 3 / 7 | 0.479 |
| ECOG PS | 0-1 / 2-4 | 35 / 8 | 27 / 3 | 8 / 5 | **0.041** | 26 / 7 | 9 / 1 | 0.656 |
| Smoking | Yes / No | 18 / 25 | 13 / 17 | 5 / 8 | >0.999 | 13 / 20 | 5 / 5 | 0.717 |
| Disease stage | IV / Ope rec. | 26 / 17 | 21 / 9 | 5 / 8 | 0.088 | 19 / 14 | 7 / 3 | 0.714 |
| Mutation | Del 19 / L858R | 32 / 11 | 23 / 7 | 9 / 4 | 0.708 | 24 / 9 | 8 / 2 | >0.999 |
| EGFR-TKI | Afatinib / Gefitinib or Erlotinib | 29 / 14 | 22 / 8 | 7 / 6 | 0.291 | 22 / 11 | 7 / 3 | >0.999 |

Abbreviations: VEGF, vascular endothelial growth factor; VEGFR2, vascular endothelial growth factor receptor 2; ECOG PS. eastern cooperative oncology group; Ope rec. recurrence after operation; EGFR-TKI, epidermal growth factor receptor-tyrosine kinase inhibitor.
