# Supplementary material for: Clinicopathological impact of VEGFR2 and VEGF‐C in patients with EGFR ‐major mutant NSCLC receiving osimertinib
Source: Thorac Cancer. 2023 Aug 22;14(29):2950–61. doi: 10.1111/1759-7714.15082 (PMC10569903; doi:10.1111/1759-7714.15082)
Supplement: Supplementary file 2 — Table A2. Comparison of patient demographics between Osi‐group and Control group. [file TCA-14-2950-s003.docx]

**Table A2. Comparison of patient’s demographics between Osi-group and Control group**

| Different variables | | Osi-group | Control group | *p*-value |
| --- | --- | --- | --- | --- |
|  |  | N=76 | N=43 |  |
| Age | <75 / ≥75yrs | 48 / 28 | 30 / 13 | 0.548 |
| Gender | Male / Female | 37 / 39 | 18 / 25 | 0.566 |
| ECOG PS | 0-1 / 2-4 | 64 / 12 | 35 / 8 | 0.799 |
| Smoking | Yes / No | 30 / 46 | 18 / 25 | 0.847 |
| Disease stage | IV / Ope rec. | 59 / 17 | 26 / 17 | 0.058 |
| Mutation | Del 19 / L858R | 39 / 37 | 32 / 11 | **0.019** |
| VEGFR2 | High / Low | 50 / 26 | 30 / 13 | 0.689 |
| VEGF-C | High / Low | 39 / 37 | 33 / 10 | **0.006** |

Abbreviations: VEGF, vascular endothelial growth factor; VEGFR2, vascular endothelial growth factor; ECOG PS, eastern cooperative oncology group; Ope rec. recurrence after operation.
