# Supplementary material for: Clinicopathological impact of VEGFR2 and VEGF‐C in patients with EGFR ‐major mutant NSCLC receiving osimertinib
Source: Thorac Cancer. 2023 Aug 22;14(29):2950–61. doi: 10.1111/1759-7714.15082 (PMC10569903; doi:10.1111/1759-7714.15082)
Supplement: Supplementary file 3 — Table A3. Efficacy according to VEGFR2 and VEGF‐C expression. [file TCA-14-2950-s001.docx]

**Table A3. Efficacy according to VEGFR2 and VEGF-C expression**

| Response | All patients | Del 19 | L858R | VEGFR2 | | VEFF-C | |
| --- | --- | --- | --- | --- | --- | --- | --- |
|  | (n=38) | (n=29) | (n=9) | High  (n=30) | Low  (n=8) | High  (n=31) | Low  (n=7) |
| CR | 3 | 3 | 0 | 1 | 2 | 3 | 0 |
| PR | 20 | 15 | 5 | 18 | 2 | 15 | 5 |
| SD | 6 | 5 | 1 | 5 | 1 | 5 | 1 |
| PD | 9 | 6 | 3 | 6 | 3 | 8 | 1 |
| ORR  (95% CI) | 60.5%  44.6-74.4% | 62.1%  43.9-77.3% | 55.6%  26.6-81.1% | 63.3% | 50.0% | 58.1% | 71.4% |
|  |  |  |  | *p*=0.686 | | *p*=0.680 | |
| DCR  (95% CI) | 76.3%  60.6-87.2% | 79.3%  61.2-90.5% | 66.6%  35.1-88.2% | 80.0% | 62.5% | 74.2% | 85.7% |
|  |  |  |  | *p*=0.363 | | *p*>0.999 | |

Abbreviations: CR, complete response; PR, partial response; SD, stable disease; PR, progressive disease; ORR, objective response rate; DCR, disease control rate; 95% CI, 95% confidence interval; VEGF, vascular endothelial growth factor; VEGFR2, vascular endothelial growth factor receptor 2.
