# Supplementary material for: Clinicopathological impact of VEGFR2 and VEGF‐C in patients with EGFR ‐major mutant NSCLC receiving osimertinib
Source: Thorac Cancer. 2023 Aug 22;14(29):2950–61. doi: 10.1111/1759-7714.15082 (PMC10569903; doi:10.1111/1759-7714.15082)
Supplement: Supplementary file 4 — Table A4. Univariate and multivariate analysis in patients with del19 (n = 39). [file TCA-14-2950-s002.docx]

**Table A4. Univariate and multivariate analysis in patients with del 19 (n=39)**

| Different variables | | Progression-free survival | | | | | Overall survival | | | | | |
| --- | --- | --- | --- | --- | --- | --- | --- | --- | --- | --- | --- | --- |
|  |  | **Univariate analysis** | | **Multivariate analysis** | | | **Univariate analysis** | | **Multivariate analysis** | | | |
|  |  | MST (days) | *p*-value | HR | 95% CI | *p*-value | MST (days) | *p*-value | HR | 95% CI | *p*-value |  |
| Age | <75 / ≥75yrs | 710 / 808 | 0.338 | 0.823 | 0.251-2.274 | 0.722 | 1142 / 844 | 0.603 | 2.156 | 0.624-6.558 | 0.210 |  |
| Gender | Male / Female | 710 / 808 | 0.964 | 0.680 | 0.227-1.787 | 0.446 | 1142 / 844 | 0.699 | 0.762 | 0.231-2.232 | 0.629 |  |
| ECOG PS | 0-1 / 2-4 | 808 / 206 | **0.006** | 0.295 | 0.091-0.972 | **0.045** | 1142 / 248 | **0.009** | 0.348 | 0.104-1.230 | 0.097 |  |
| Smoking | Yes / No | 710 / 808 | 0.881 |  |  |  | 1142 / 844 | 0.902 |  |  |  |  |
| CNS meta. | Yes / No | 710 / 790 | 0.703 |  |  |  | NR / 1142 | 0.461 |  |  |  |  |
| PM | Yes / No | 378 / 790 | 0.222 |  |  |  | 880 / 1267 | 0.134 |  |  |  |  |
| Pleural ca. | Yes / No | 516 / 808 | 0.413 |  |  |  | 880 / 1142 | 0.502 |  |  |  |  |
| Lever meta. | Yes / No | 937 / 710 | 0.710 |  |  |  | 1186 / 880 | 0.934 |  |  |  |  |
| Bone meta. | Yes / No | 443 / 790 | 0.615 |  |  |  | 844 / 1142 | 0.847 |  |  |  |  |
| Ki-67 LI | High / Low | 443 / 825 | 0.193 |  |  |  | 844 / 1142 | 0.102 |  |  |  |  |
| ASCT2 | High / Low | 846 / 443 | 0.178 |  |  |  | 880 / 1186 | 0.740 |  |  |  |  |
| VEGFR2 | High / Low | 412 / 825 | **0.038** | 2.181 | 0.705-8.310 | 0.183 | 717 / NR | **0.031** | 4.616 | 1.122-31.44 | **0.032** |  |
| VEGF-C | High / Low | 808 / 378 | 0.098 |  |  |  | 1297 / 717 | 0.171 |  |  |  |  |
| VEGFR2/VEGF-C | Positive / Negative | 808 / 710 | 0.952 |  |  |  | 844 / 1142 | 0.919 |  |  |  |  |

Abbreviations: VEGF, vascular endothelial growth factor; VEGFR2, vascular endothelial growth factor; ECOG PS. eastern cooperative oncology group; Ope rec. recurrence after operation; CNS, central nervous system; PM. Pulmonary metastases; meta. Metastasis; LI, labeling index; MST, median survival time; HR, hazard ratio; 95% CI, 95% confidence interval.
