# Supplementary material for: Clinicopathological impact of VEGFR2 and VEGF‐C in patients with EGFR ‐major mutant NSCLC receiving osimertinib
Source: Thorac Cancer. 2023 Aug 22;14(29):2950–61. doi: 10.1111/1759-7714.15082 (PMC10569903; doi:10.1111/1759-7714.15082)
Supplement: Supplementary file 5 — Table A5. Univariate and multivariate analysis in patients with L858R (n = 27). [file TCA-14-2950-s005.docx]

**Table A5. Univariate and multivariate analysis in patients with L858R (n=27)**

| Different variables | | Progression-free survival | | | | | Overall survival | | | | |
| --- | --- | --- | --- | --- | --- | --- | --- | --- | --- | --- | --- |
|  |  | **Univariate analysis** | | **Multivariate analysis** | | | **Univariate analysis** | | **Multivariate analysis** | | |
|  |  | MST (days) | *p*-value | HR | 95% CI | *p*-value | MST (days) | *p*-value | HR | 95% CI | *p*-value |
| Age | <75 / ≥75yrs | 482 / 228 | 0.436 | 1.154 | 0.513-2.585 | 0.724 | 785 / 605 | 0.262 | 1.179 | 0.482-2.854 | 0.712 |
| Gender | Male / Female | 566 / 339 | 0.602 | 0.954 | 0.424-2.244 | 0.911 | 826 / 723 | 0.300 | 1.028 | 0.385-3.037 | 0.957 |
| ECOG PS | 0-1 / 2-4 | 549 / 215 | 0.114 | 0.617 | 0.231-1.975 | 0.388 | 826 / 258 | **<0.001** | 0.302 | 0.093-1.074 | 0.063 |
| Smoking | Yes / No | 549 / 339 | 0.856 |  |  |  | 785 / 746 | 0.729 |  |  |  |
| CNS meta. | Yes / No | 444 / 416 | 0.347 |  |  |  | 753 / 826 | 0.437 |  |  |  |
| PM | Yes / No | 416 / 444 | 0.646 |  |  |  | 753 / 811 | 0.616 |  |  |  |
| Pleural ca. | Yes / No | 377 / 549 | 0.975 |  |  |  | 769 / 753 | 0.459 |  |  |  |
| Lever meta. | Yes / No | 243 / 566 | 0.107 |  |  |  | 552 / 811 | 0.089 |  |  |  |
| Bone meta. | Yes / No | 339 / 482 | 0.397 |  |  |  | 769 / 605 | 0.579 |  |  |  |
| Ki-67 LI | High / Low | 377 / 549 | 0.635 |  |  |  | 557 / 769 | 0.981 |  |  |  |
| ASCT2 | High / Low | 580 / 339 | 0.518 |  |  |  | 785 / 769 | 0.515 |  |  |  |
| VEGFR2 | High / Low | 568 / 271 | 0.700 |  |  |  | 769 / 723 | 0.893 |  |  |  |
| VEGF-C | High / Low | 338 / 568 | 0.187 |  |  |  | 605 / 988 | 0.093 |  |  |  |
| VEGFR2/VEGF-C | Positive / Negative | 271 / 566 | **0.016** | 2.403 | 1.027-5.721 | **0.043** | 605 / 976 | **0.009** | 2.641 | 0.992-7.367 | 0.051 |

Abbreviations: VEGF, vascular endothelial growth factor; VEGFR2, vascular endothelial growth factor; ECOG PS. eastern cooperative oncology group; Ope rec. recurrence after operation; CNS, central nervous system; PM. Pulmonary metastases; meta. Metastasis; LI, labeling index; MST, median survival time; HR, hazard ratio; 95% CI, 95% confidence interval.
